# Comparison of supine and beach-chair positions in antegrade intramedullary nailing of humeral shaft fractures: a retrospective study on surgery duration and fluoroscopy exposure

**DOI:** 10.1007/s00590-025-04621-w

**Published:** 2025-12-26

**Authors:** Katharina Kirchhoff, Christina Zeiner, Daniel P. Berthold, Georg Siebenbürger, Markus Bormann, Fabian Gilbert

**Affiliations:** 1 Department of Orthopaedic and Trauma Surgery, Musculoskeletal University Center Munich (MUM), LMU Klinikum, Munich, Germany; 2 Orthocenter Munich, Munich, Germany; 3 Department of Orthopaedic and Trauma Surgery, Kreisklinik Ebersberg, Ebersberg, Germany

**Keywords:** Patient positioning, Humeral shaft fracture, Surgical duration, Antegrade intramedullary nailing, Beach-chair position, Supine position

## Abstract

**Purpose:**

This study aims to compare the duration of surgery and fluoroscopy exposure between the supine and beach-chair positions during antegrade intramedullary nailing (IMN) of humeral shaft fractures.

**Methods:**

A retrospective, single-center study was conducted at a German Level I Trauma Center from January 2021 to August 2024. Patients included were aged 18–95 with confirmed humeral shaft fractures diagnosed via radiographic imaging. Exclusion criteria comprised fractures older than two weeks, polytrauma cases, concomitant neurovascular injuries, and patients undergoing alternative osteosynthesis procedures during the same surgery. Surgery was performed in either the supine or beach-chair position, and comparisons were made regarding operative duration, fluoroscopy time, and radiation dose exposure. Furthermore, the influence of fracture complexity (A-,B- and C- fractures according to AO) on these three parameters was tested. Demographic data were also collected and analyzed.

**Results:**

A total of 79 patients were initially considered, with 59 meeting the inclusion criteria after the exclusion of 20 polytrauma cases. The final cohort consisted of 25 males (42.4%) and 34 females (57.6%), with a mean age of 67.1 years (± 20.1) and a mean BMI of 25.6 (± 5.6). Surgical outcomes demonstrated that procedures performed in the beach-chair position were significantly longer (103.5 ± 54.0 min) compared to those in the supine position (89.0 ± 42.0 min, *p* = 0.023). Furthermore, radiation exposure was considerably lower in the supine position (1,0 centigray/cm^2^ ± 65,6) compared to the beach-chair position (161.4 centigray/cm^2^ ± 245.1, *p* < 0.001). Although fluoroscopy duration was slightly longer in the beach-chair position (4.0 ± 4.2 min) than in the supine position (3.0 ± 2.6 min), this difference was not statistically significant. Neither surgery time (*p* = 0.855), nor fluoroscopy duration (*p* = 0.726), nor fluoroscopy dose (*p* = 0.052) reached a statistically significant difference in surgeries for A, B and C fractures.

**Conclusion:**

Supine positioning for antegrade IMN of humeral shaft fractures significantly reduces both operative duration and radiation exposure compared to the beach-chair position, whereas fracture complexity does not significantly influence these parameters. The findings suggest that the supine position offers a viable alternative for patient positioning in these procedures.

## Introduction

Fractures of the humeral shaft, predominantly resulting from high-energy trauma, account for approximately 3–5% of all fractures and comprise nearly one-third of osteoporotic fractures [1, 2]. This type of fracture is most frequently observed in two distinct age groups: individuals aged 20–30 years and those over 60 years [3]. While non-operative treatment remains the standard in many fracture patterns— employing functional bracing or hanging arm casts—surgical intervention still remains a frequently utilized approach, especially in complex fractures with severe fragment displacement [1, 4].

Among surgical techniques, intramedullary nailing (IMN) is a commonly employed method for managing humeral shaft fractures. This technique is favored by some authors due to its capacity to provide rapid pain relief, immediate stabilization that facilitates early mobilization, effective fracture alignment, and accelerated recovery by preserving periosteal blood supply, which is critical for bone healing [5–7].

Depending on the fracture pattern, management strategies may vary between open reduction with plate osteosynthesis and closed reduction with IMN. However, neither technique has conclusively demonstrated superiority over the other to date [8–11]. Consequently, the choice of technique is often influenced by the surgeon’s expertise, preference, and the fracture’s specific characteristics.

Cohort studies have consistently reported favorable clinical outcomes, with approximately 90% of patients achieving good to excellent results following intramedullary nailing for humeral shaft fractures [3, 12, 13].

Patient positioning during IMN plays a pivotal role in achieving optimal surgical outcomes and minimizing postoperative complications. The two most commonly employed positions for this procedure are the beach-chair position (BCP) and the supine position. Despite the importance of positioning, there is a limited body of literature specifically comparing surgical duration and radiation exposure associated with these two approaches in humeral IMN procedures.

This retrospective study was designed to evaluate the impact of patient positioning—specifically the supine and beach-chair positions—on key intraoperative parameters such as surgical duration and radiation exposure. By analyzing these factors, the study aims to provide valuable insights into optimizing procedural efficiency and enhancing perioperative outcomes in the management of humeral shaft fractures using antegrade intramedullary nailing (IMN).

## Methods

### Patient selection

A single-center retrospective study was conducted at a German Level I Trauma Center between January 1, 2021, and August 8, 2024, involving patients who underwent percutaneous antegrade intramedullary nailing (IMN) for the management of humeral shaft fractures. Eligible patients presented with isolated closed trauma and radiographically confirmed displaced humeral shaft fractures.

Patients were excluded if they had:

- Fractures exceeding two weeks in age,
- Polytrauma cases,
- Concomitant neurovascular injuries, or.
- Concurrent osteosynthesis procedures performed alongside IMN during the same surgical intervention.

Data collection was performed by an independent observer utilizing the institution’s digital database to ensure unbiased reporting. Recorded variables included patient demographics (gender, age at surgery, and body mass index [BMI]) and intraoperative patient positioning.

Operative time was defined as the interval between the initial skin incision and the completion of wound closure. As per institutional protocol, fluoroscopy parameters—including fluoroscopy duration and radiation dose—were obtained directly from the C-arm display and meticulously documented.

Preoperative imaging included standardized anteroposterior (ap) and Y-view X-rays of the shoulder and elbow to assess fracture morphology and alignment. All fractures were classified in accordance with the established Arbeitsgemeinschaft für Osteosynthesefragen (AO) classification system to ensure consistent fracture characterization.

### Surgical technique

All surgical procedures were performed within three days post-trauma by board-certified orthopedic surgeons. The surgical procedures in each cohort (beach-chair and supine position) were performed by surgeons with comparable levels of expertise. Each group consisted of two senior shoulder surgeons with over ten years of experience in orthopaedic trauma surgery with focus on shoulder and elbow pathologies as well as three other board-certified trauma surgeons who were actively engaged in subspeciality training in shoulder surgery.

Each patient underwent general anesthesia combined with an interscalene block. Patient positioning was determined at the discretion of the operating surgeon, independent of the fracture morphology. Intraoperative imaging was conducted under fluoroscopic guidance throughout the procedure. The primary distinction lies in the patient positioning technique. In the supine position, the procedure is performed on a carbon table, with the patient`s arm supported alongside the torso on the table. Fracture reduction is primarily achieved through longitudinal traction, and ap view is easily reproducible due to the C-arm being placed in a neutral position. Controversely, during beach-chair positioning, a conventional shoulder table is utilized (the same one that is employed in arthroscopic procedures). Here, the C-arm must be tilted to match the inclination of the patient`s torso to obtain an accurate ap view. Only closed reduction techniques were used in this study, no mini incisions at the fracture site were conducted and no cerclage technique was used.

### Beach-chair-positioning

**Fig. 1:**
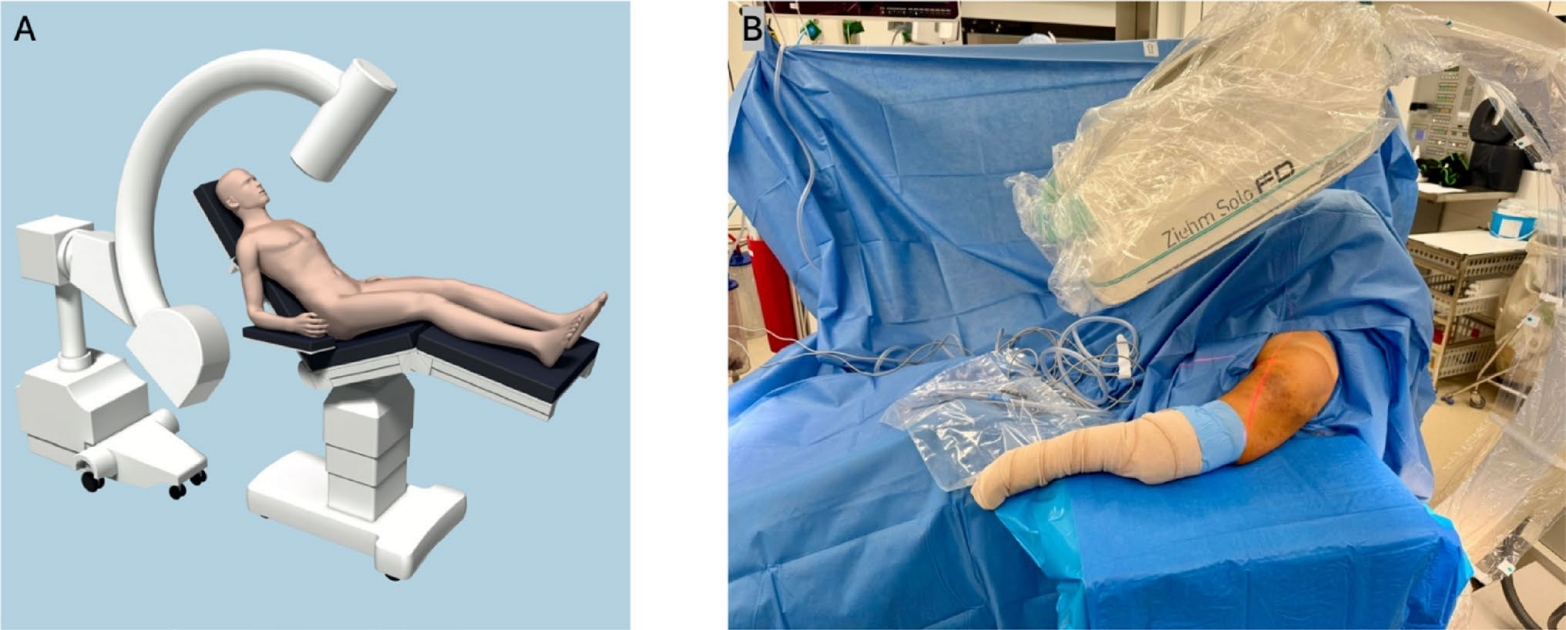
**A** Scheme of patient beach-chair position (used with permission from DePuy Synthes, Switzerland). **B** Image of beach-chair position with patient`s torso elevated to an angle of 40° and C-arm inclined by 40°

In this position, the patient was seated upright at a 40° incline, using a standard table (Jupiter, Trumpf Medical Systems) The affected arm was positioned freely at the lateral edge of the operating table, allowing unrestricted movement. The C-arm was placed in 40° inclination for an ap view of the proximal humerus. The arm was gently reclined with the forearm supported on an adjustable arm table. This positioning enabled fracture reduction via longitudinal traction and gentle retroversion of the humerus, facilitating optimal alignment of the fracture fragments (Fig. 1).

### Supine-positioning

**Fig. 2:**
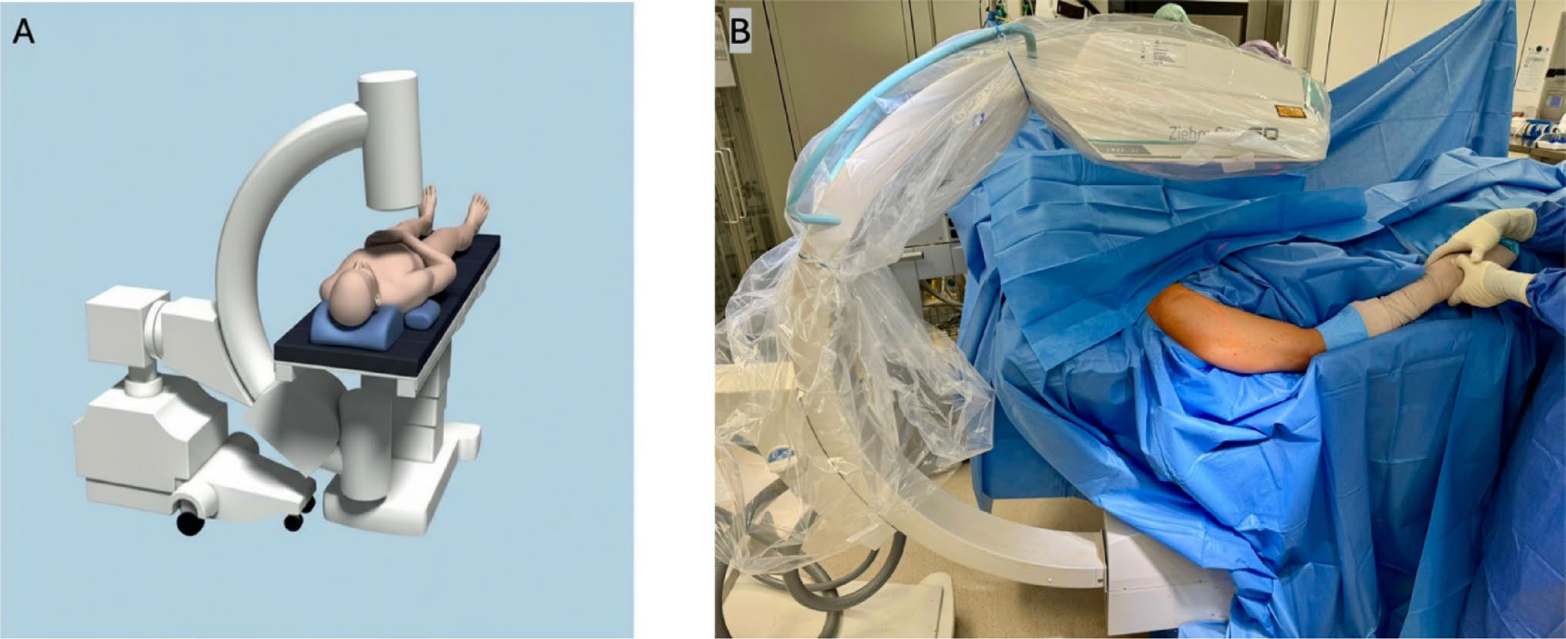
**A** Scheme of patient supine position (used with permission from DePuy Synthes, Switzerland). **B** Image of supine position with neutral position of C-arm (no inclination)

For the supine position, the patient was placed at the lateral edge of a radiolucent carbon operating table (TruSystem 7500 Carbon Float Line, Trumpf Medical Systems) to ensure unobstructed access to the surgical site and optimal fluoroscopic visualization. The C-Arm was placed perpendicular to the longitudinal axis of the humeral shaft in the ap view. Reduction was obtained via longitudinal traction and lateral or medial external pressure on the fracture site (Fig. 2).

### Implantation of the IMN

The implant utilized in all cases was the MultiLoc Humeral Nail® (DePuy Synthes, Switzerland). After performing a anterolateral approach the entry point for nail insertion was meticulously identified near the apex of the humeral head, ensuring alignment with the humeral axis. In some cases, access to the apex required the use of Kirschner wires used as joysticks. A longitudinal incision of 1–2 cm was made through the supraspinatus tendon to position the guide rod and the medullary canal was then opened with a drill bit. If fracture reduction over the reaming rod was not possible, the reduction instrument for MultiLoc Humeral Nailing System (DePuy Synthes, Switzerland) was inserted into the medullary canal. After nail insertion, proximal and distal locking were achieved using self-stabilizing screws to enhance construct stability.

The implantation procedure adhered strictly to the manufacturer’s technical guidelines. Accurate fracture reduction was confirmed intraoperatively using standardized anteroposterior (AP) and lateral fluoroscopic views to verify alignment and ensure correct implant positioning.

### Statistical analysis

Quantitative variables with a normal distribution were presented as mean and standard deviation (SD) while qualitative variables were expressed as counts (N) and percentages (%). However, some quantitative variables, namely the fluoroscopy dose, the fluoroscopy duration and the surgery time were described using median values and interquartile ranges.To assess the distribution pattern of key metric variables such as fluoroscopy dose, surgery duration, fluoroscopy duration, age and BMI, the Shapiro–Wilk test was applied. A normal distribution was confirmed only for surgery duration and BMI in the supine position, along with the surgical duration, fluoroscopy dose, and radiation dose in C fractures. Statistical comparisons for non-normally distributed variables employed the Mann–Whitney U test for two-group analyses (beach-chair vs. supine) and the Kruskal–Wallis test was used for three-group comparisons (A-,B-, and C-fractures). For categorical variables such as gender, side of surgery and occurring postoperative complications, the Chi^2^-test was used.

A significance threshold of *p* < 0.05 was established, with results below this threshold deemed statistically significant. All statistical analyses were performed using IBM SPSS for Windows (Version 30.0.0) (SPSS Inc., Chicago, Illinois) and Excel® 2019 (Microsoft Office, Microsoft, USA).

## Results

### Demographics

During the study period, a total of 79 patients presenting with humeral shaft fractures underwent treatment with antegrade intramedullary nailing (IMN) at the authors’ institution. Following the exclusion of 20 patients due to polytrauma-related conditions, a final cohort of 59 patients was included in the analysis.

The whole study population comprised 25 males (42.4%) and 34 females (57.6%). The mean body mass index (BMI) was 25.6 while the mean age was 67.1 years.

In terms of fracture laterality, the left humeral shaft was affected in 36 cases (61.0%), while the right humeral shaft was involved in 23 cases (39.0%).

Regarding patient positioning during surgery, 17 patients (28.8%) underwent IMN in the supine position, while 42 patients (71.2%) were treated in the beach-chair position.

Between both surgical positions BMI, age and gender distribution showed no statistically significant difference and were comparable. However, there was a significant difference regarding the side of the affected arm, as 58.8% of patients in the supine position were operated on the right arm, compared to 31.0% of patients in the beach-chair position.

Detailed demographic data are presented in Table 1.

**Table 1:** Demographic and surgical data stratified by patient positioning: All data could be collected retrospectively except for the BMI of one patient in the beach-chair group and the fluoroscopy duration of two surgeries in the beach-chair group. Fluoroscopy duration, fluoroscopy dose and surgery duration were described using medians and interquartile ranges. All other metric variables were described using mean values and standard deviations

Parameter	Total (N = 59)	Supine (N = 17)	Beach-Chair (N = 42)	*p*-Value
Age	67.1 ± 20.1	65.9 ± 23.3	67.6 ± 18.9	0.913
Males Females	25 (42.4%)	6 (35.3%)	19 (45.2%)	0.484
	34 (57.6%)	11 (64.7%)	23 (54.8%)	
Left side Right side	36 (61.0%)	7 (41.2%)	29 (69.0%)	0.047
	23 (39.0%)	10 (58.8%)	13 (31.0%)	
BMI (kg/m^2^)	25.6 ± 5.5	24.9 ± 4.9	25.8 ± 5.8	0.797
Surgery duration (minutes)	96.0 ± 43.0	89.0 ± 42.0	103.5 ± 54.0	0.023
Fluoroscopy dose (centigray/cm^2^)	82.9 ± 221.3	1.0 ± 65.6	161.4 ± 245.1	< 0.001
Fluorscopy duration (minutes)	3.6 ± 3.3	3.0 ± 2.6	4.0 ± 4.2	0.519
AO-classification A-fractures B-fractures C-fractures	43 (72.9%) 9 (15.3%) 7 (11.9%)	12 (70.6%) 3 (17.6%) 2 (11.8%)	31 (73.8%) 6 (14.3%) 5 (11.9%)	0.948

### Classifications

Among all the patients (N = 59) included in the study, 72.9% were classified as AO type 12-A fractures, reflecting a predominance of simple fracture patterns within the study cohort. Conversely, complex fractures categorized as AO type 12-C were identified in 11.9% of the patient population.

**Fig. 3:**
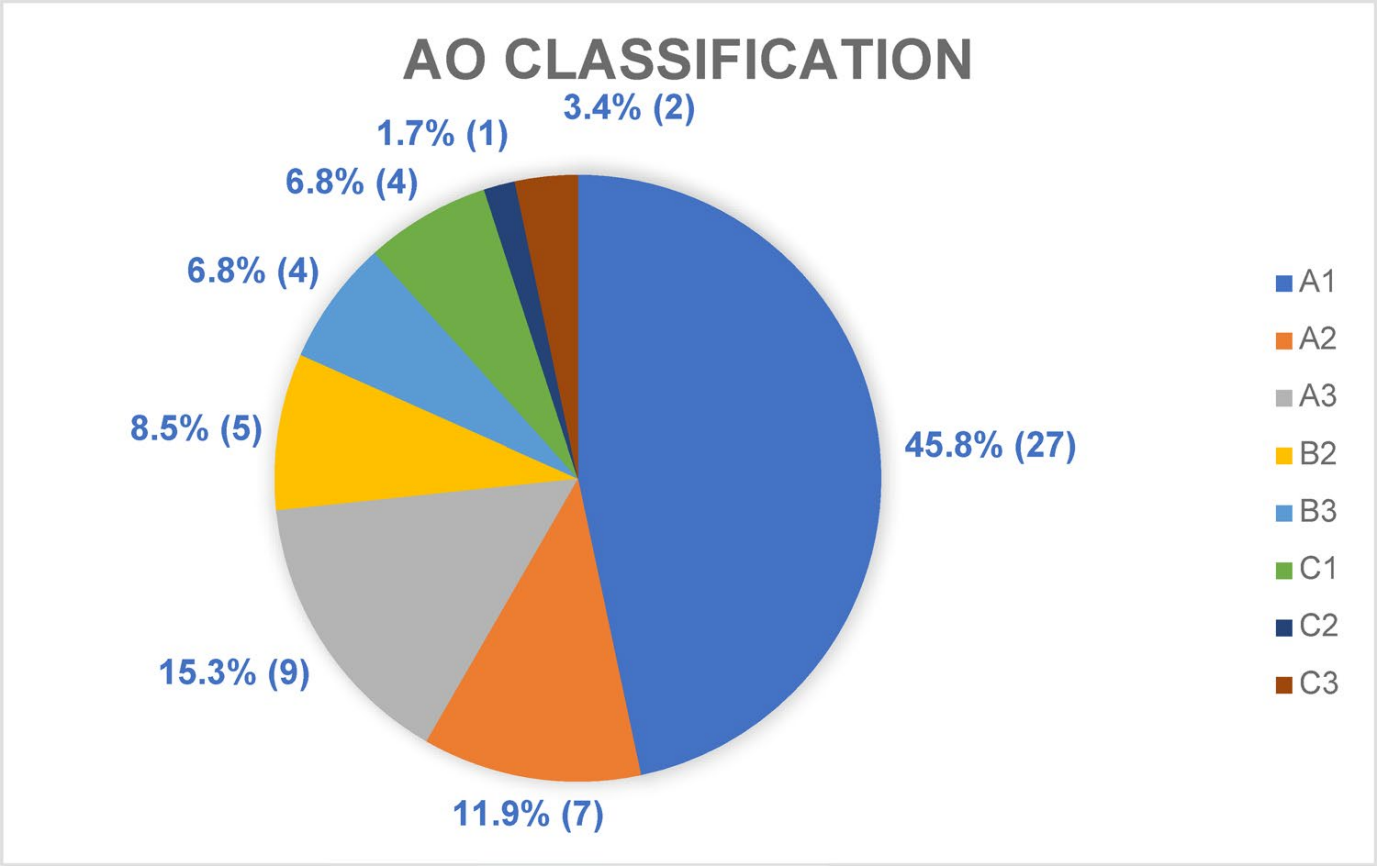
The distribution of humeral shaft fractures within the study cohort (n = 59) was categorized according to the Arbeitsgemeinschaft für Osteosynthesefragen (AO) classification system

Comprehensive details regarding fracture classification are illustrated in Fig. 3.

There were no statistically significant differences regarding the complexity of operated fractures between both groups (*p* = 0.948). Further information can be found in Table 1.

### Surgical and fluoroscopy duration

#### Comparison by patient positioning

Operative procedures conducted in the beach-chair position demonstrated a significantly longer median duration of 103.5 min compared to those performed in the supine position, which had a median duration of 89.0 min (*p* = 0.023).

A statistically significant difference was also observed in the median fluoroscopy dose. Patients positioned supine received a mean radiation dose of 1.0 centigray/cm^2^, which was considerably lower than the median dose of 161.4 centigray/cm^2^ recorded in the beach-chair position. (*p* < 0.001).

Although the median fluoroscopy duration was longer in the beach-chair position (4.0 min) than in the supine position (3.0 min), this difference did not reach statistical significance (*p* = 0.519). More detailed information can be found in Table 1.

### Comparison by fracture complexity

The comparison across A, B and C fractures according to the AO classification revealed no statistically significant differences in fluoroscopy duration (*p* = 0.726), radiation dose (*p* = 0.052) or surgery time (*p* = 0.855). The median fluoroscopy duration (3.5 vs. 3.8 vs. 4.2 min) was almost equally high for all three fracture types. The median surgery durations for B-type fractures was 112.0 min, which exceeded the median durations for A-type (95.0 min) and C-type fractures (100.0 min). However, the fluoroscopy dose with median values of 81.7 centigray/cm^2^ for A-fractures, 1.1 centigray/cm^2^ for B-fractures and 241.3 centigray/cm^2^ for C-fractures were statistically not significant. More detailed information can be found in Table 2.

**Table 2:** Surgical data stratified by fracture complexity: All data could be collected retrospectively except for the fluoroscopy duration of two A-fracture surgeries. The data is presented using medians and interquartile ranges

	A-fractures (N = 43)	B-fractures (N = 9)	C-fractures (N = 7)	*p*-Value
Fluoroscopy duration (minutes)	3.5 ± 4.3	3.8 ± 2.9	4.2 ± 2.7	0.726
Fluoroscopy dose (centigray/cm^2^)	81.7 ± 196.4	1.1 ± 173.5	241.3 ± 127.6	0.052
Surgery duration (minutes)	95.0 ± 43.0	112.0 ± 64.0	100.0 ± 47.0	0.855

### Complications

In 79.7% (N = 47) of all cases included in the study, there were no complications reported, leaving 20.3% (N = 12) with postoperative complications. While 41.2% (N = 7) from the supine position suffered from complications, 11.9% (N = 5) from the beach-chair position had postoperative problems, which was a statistically significant difference (*p* = 0.011).

The most frequently documented problem was the fragment displacement of less than 0.5 cm, which happened after surgery in supine position (N = 3) as well as beach chair position (N = 2). However, surgical revision was not necessary in any of the cases because of proper alignment and bone healing after 8 weeks.

In the supine position one patient suffered from postoperative CRPS, one patient from a distal locking screw loosening after three months because of the callus, one patient from a soft tissue infection after 8 weeks and one patient from a displacement of the distal locking screw after 6 months due to osteoporosis. The soft tissue infection was resolved after debridement and oral antibiotics therapy for one week. The dislocated locking screw was removed, while the loosened locking screw was not removed.

In the beach- chair position there was one reported case of reduced abduction after six months due to defect arthropathy, one case of subacromial impingement and one case of cutting-out of the nail. The patient with the subacromial impingement and the patient with the cutting-out received an implant removal. Further details can be found in Table 3.

**Table 3:** Postoperative complications

Complications	Total (N = 59)	Supine (N = 17)	Beach-Chair (N = 42)	Consequence	*p*-Value
Total	12 (20.3%)	7 (41.2%)	5 (11.9%)	–	0.011
Fragment displacement	5 (8.5%)	3 (17.7%)	2 (4.8%)	No removal or reoperation	
CRPS^1^	1 (1.7%)	1 (5.9%)	1 (2.4%)	–	
Subacromial impingement	1 (1.7%)	–	1 (2.4%)	Implant removal	
Cut-out of nail	1 (1.7%)	–	1 (2.4%)	Implant removal	
Locking screw loosening	1 (1.7%)	1 (5.9%)	–	No removal	
Locking screw displacement	1 (1.7%)	1 (5.9%)	–	Locking screw removal	
Superficial soft tissue infection	1 (1.7%)	1 (5.9%)	–	Debridement and antibiotics (7 days)	
Defect arthropathy	1 (1.7%)	–	1 (2.4%)		

^1^complex regional pain syndrome

## Discussion

The key finding of this study is that supine positioning during osteosynthesis of humeral shaft fractures utilizing antegrade intramedullary nailing (IMN) significantly reduces both operative duration and radiation exposure when compared to the beach-chair position. Notably, surgical procedures performed in the supine position were, on average, 25 min shorter, and the associated radiation dose was markedly lower. Moreover, surgery and fluoroscopy duration were almost equally high across surgeries for A-, B-and C- fractures and although the average radiation dose in C-fractures was higher than in less complex fracture types, this difference did not reach statistical significance. Altogether these results emphasize the supine position as a more efficient and potentially safer alternative for both patients and surgical staff.

To circumvent the need for prone or lateral decubitus positioning during surgery, a Korean study introduced the use of retrograde IMN in the supine position for humeral fractures. The authors highlighted that this positioning strategy is particularly advantageous for patients with head injuries, facial trauma, spinal injuries, or multiple trauma, as it minimizes the risk of exacerbating pre-existing conditions [14].

Although there are limited studies directly examining the effect of patient positioning on radiation exposure during IMN procedures, previous research has explored related parameters. A study conducted by Igrek et al. investigated the impact of C-arm configuration (inverted versus standard-horizontal positioning) on surgical duration and fluoroscopy exposure in pediatric supracondylar humeral fractures. All patients in their study underwent closed reduction and percutaneous K-wire pinning. The authors observed that utilizing a biplanar C-arm configuration reduced radiation exposure to the surgeon’s neck area and was associated with a shorter fluoroscopy duration. However, this method extended the total surgical time due to the additional steps required to transition the C-arm from a vertical to a horizontal orientation [15].

An additional strategy to mitigate both operative time and radiation exposure was proposed by Erdogan et al. Their approach involved preoperative skin marking to delineate the anatomical boundaries of the humerus prior to performing closed reduction and percutaneous pinning of supracondylar humeral fractures. This method enhanced the surgeon’s three-dimensional spatial awareness, which resulted in significantly reduced operative duration and radiation exposure [16].

Collectively, these findings align with the current study’s conclusion that the supine position offers notable advantages in reducing both surgical duration and radiation exposure, positioning it as a favorable alternative to the beach-chair position for patients undergoing IMN for humeral shaft fractures.

The findings from this study demonstrate that the supine position facilitates shorter operative durations by enabling a more controlled, ergonomic, and safe approach to fracture reduction and intraoperative handling in our clinical setting. Under fluoroscopic guidance, the intramedullary nail (IMN) is inserted into the proximal humeral canal, traverses the fracture site, and is then advanced distally.

Precise rotational alignment of the humerus is manually achieved through careful manipulation. Key indicators for correct torsional alignment include:

- Radiographic visualization of the humeral head in the anteroposterior (AP) view,
- A homogeneous cortical thickness transition between the proximal and distal humeral segments, and.
- The clinical alignment of the elbow and forearm.

By aligning these anatomical landmarks parallel to the operating table, an optimal AP view under C-arm fluoroscopy can be readily obtained. Additionally, the application of continuous longitudinal traction to the distal arm facilitates simplified fracture reduction, which may be further enhanced through controlled lateral or medial stress.

In addition to our findings favoring the supine position, the lateral decubitus position, as described by Barret et al., has been identified as another viable alternative. Compared to the beach-chair position, the lateral position offers a more centralized entry point, which enhances the precision of nail insertion and may contribute to improved clinical outcomes [14–17].

Beyond technical considerations, the beach-chair position (BCP) has been associated with adverse hemodynamic effects. A study by Jeong et al. reported a 10% reduction in cerebral oxygen saturation in patients undergoing arthroscopic shoulder surgery in the BCP under general anesthesia. This desaturation was reversible upon patient repositioning [18].

Further research has linked the BCP to impaired cerebral autoregulation, reflected by higher cerebral oximetry values and lower regional cerebral oxygen saturation when compared to the lateral decubitus position [19]. Similarly, McCulloch et al. reported that relative hypotension during BCP procedures under general anesthesia may lead to regional cerebral oxygen desaturation, emphasizing the potential risks of inadequate cerebral perfusion in this position [20].

In addition, Cho et al. investigated the administration of arginine vasopressin during arthroscopic shoulder surgery in the BCP. While this intervention effectively prevented hypotension, it paradoxically impaired cerebral oxygenation, reinforcing the necessity for vigilant hemodynamic monitoring and careful management of oxygen saturation levels in patients positioned in the BCP [21].

These studies underscore the importance of strict hemodynamic management to mitigate the risk of cerebral desaturation events and maintain cerebral perfusion stability, particularly in procedures requiring the BCP.

Despite its valuable findings, this study has several limitations. First, the retrospective study design precluded randomization, potentially introducing selection bias influenced by surgeon preferences. Additionally, the relatively small sample size may have reduced the statistical power of the analysis, increasing the risk of a Type II error. Consequently, further investigations involving larger cohorts are warranted to confirm these results and provide more comprehensive clinical insights.

Moreover, this study did not evaluate clinical outcomes, long-term functional results, or comparative radiological findings. Lastly, it is important to acknowledge that operative duration and surgical outcomes can be significantly influenced by the experience and technical proficiency of the operating surgeon, which varied among the participating surgeons in this study.

Future research should aim to incorporate prospective, randomized controlled trials with larger sample sizes to better evaluate clinical outcomes, long-term success rates, and potential complications associated with different patient positioning strategies in humeral IMN procedures.

## Conclusion

This consecutive series of intramedullary nailing (IMN) procedures for the treatment of humeral shaft fractures demonstrated that positioning patients in the supine position resulted in a significant reduction in operative duration compared to the beach-chair position. Furthermore, the supine position was associated with markedly lower radiation exposure for both patients and surgical staff, while fluoroscopy duration was also reduced. Fracture complexity, however, does not significantly influence radiation exposure and surgery time.

These findings highlight the supine position as a practical, efficient, and safer intraoperative positioning strategy in the context of humeral IMN procedures. Its advantages in reducing both operative time and radiation exposure support its consideration as a preferred positioning technique in appropriate clinical scenarios.

## Data Availability

This published article contains all the data generated or analyzed during this study.

## Abbreviations

AO: Arbeitsgemeinschaft für osteosynthesefragen
AP: Anteroposterior
BCP: Beach-chair position
BMI: Body mass index
CRPS: Complex regional pain syndrome
IMN: Intramedullary nailing
